# Santé sexuelle et reproductive des adolescentes de Bobo Dioulasso, Burkina Faso: place de la communication parents-adolescentes pour la réduction des risques

**Published:** 2012-04-10

**Authors:** Donmozoun Télesphore Some, Der Adolphe Some, Hervé Hien, Ramata Diallo, Dézémon Zingue, Ibrahim Diallo, Serge Diagbouga, Blami Dao

**Affiliations:** 1 Centre Muraz, Avenue Mamadou Konaté, porte 2006, BP 390, Bobo Dioulasso, Burkina Faso; 2 Centre Hospitalier Universitaire Sanou Souro, 01 BP 3482 Bobo Dioulasso, Burkina Faso

**Keywords:** Communication, santé de la reproductive, adolescentes, Burkina Faso

## Abstract

**Introduction:**

Les adolescentes sont très vulnérables face aux Infections Sexuellement Transmissibles (IST) et au VIH/SIDA. Notre étude a pour objectifs d’explorer la qualité de la communication entre les adolescentes et leurs parents sur les IST/VIH/SIDA et de recueillir leurs suggestions pour l’amélioration de cette communication.

**Méthodes:**

L’étude était transversale qualitative sur 2 mois. La population de l’étude était composée par des adolescentes de 13 à 17 ans et leurs parents habitant la ville de Bobo Dioulasso. Vingt (20) adolescentes scolarisées ont été tirés au sort dans trois établissements secondaires et 20 autres non scolarisées choisies de façon accidentelle dans la ville. Les informations ont été collectées à l’aide de 8 focus groups. Les discussions ont été enregistrées sur cassettes, retranscrites en verbatim, et analysées à l’aide du logiciel QSR NVIVO 2.0.

**Résultats:**

Les adolescentes et leurs parents communiquent très peu ou pas sur les questions des IST/VIH/SIDA; l’auto-information par les paires ou les médias est la première source d’information. Pour les parents, l’information existe et est accessible aux adolescentes alors que pour ces dernières, leurs connaissances de ces maladies sont parfois erronées. L’abstinence, la fidélité et le dépistage volontaire comme moyen de prévention sont très peu évoqués par les adolescentes de même que par leurs parents.

**Conclusion:**

La communication parents-adolescentes est insuffisante voire absente dans certains milieux. Il est urgent de conduire des actions auprès des adolescentes et leurs parents pour mieux les informer sur les IST/VIH/SIDA et améliorer la communication sur ces maladies.

## Introduction

Les adolescentes courent un risque majeur et immédiat de contracter une infection sexuellement transmissible(IST), le VIH/sida ou une grossesse non désirée [1, 2]; cette situation est préoccupante pour de nombreux états. Malgré les efforts d’information et de sensibilisation que ces états développent, les comportements sexuels à risque des adolescentes ne semblent pas s’améliorer. Les parents comme maillon dans l’éducation d’une sexualité responsable de la jeune fille ont souvent été très peu sollicités. De nombreuses études montrent la nécessité de l’implication des parents dans l’étude à la santé de la reproduction des jeunes et adolescents. Paradoxalement, pour de nombreux parents, les pesanteurs socioculturelles font du sexe un tabou. Parler de sexualité, surtout avec des adolescentes devient un exercice difficile. Alors que ces jeunes de nos jours, ont un besoin de cette communication [3, 4].

Selon les auteurs, la communication entre les parents et les adolescentes est une stratégie qui serait des plus efficaces si elle était vraiment mise en pratique. L’absence de communication parents-adolescentes sur la sexualité conduit celles-ci à des rapports sexuels précoces, au multi partenariat, à des avortements clandestins et à une non-utilisation de préservatifs [5–7].

Quelques études menées au Burkina Faso montrent qu’il ya un grand déficit de communication entre les parents (surtout les pères) et les adolescentes. Les principales causes retrouvées chez les parents sont la crise de l’autorité parentale, leur démission, l’agressivité ou la violence envers les adolescentes. Quant aux adolescentes, elles ont peur des parents, ou leur manquent de respect et d’obéissance [8]. Les connaissances des adolescentes sont limitées sur les méthodes contraceptives modernes; environ 60% des adolescents ne connaissent pas la période féconde dans le cycle menstruel [9].

Ce travail a pour objectifs de recenser les thèmes de discussion des adolescentes en rapport avec les IST/VIH/SIDA et GND, de déterminer leurs confidents et de recueillir leurs perceptions, expériences et connaissances personnelles sur ces comportements à risqué.

## Méthodes

Il s’agit d’une étude qualitative prospective descriptive menée à Bobo Dioulasso, au Burkina Faso du 29 avril 2009 au 28 juin 2009. La population cible se composait d’adolescentes âgées de 13 à 17 ans de même que leurs parents ou tuteurs, ayant donné leur accord pour participer à l’étude. Nous avons retenu au total 40 adolescentes dont 20 régulièrement scolarisées 20 non scolarisées.

Les scolarisées ont été recrutées sur la base des listes de présence des filles de la tranche d’âge souhaitée dans 3 lycées de la ville: Lycée Ouezzin Coulibaly, Collège Sainte Marie de Tounouma, Lycée Yahvé Jiré. Elles ont été choisies à partir d’un tirage aléatoire. Les non-scolarisées ont été recrutées dans les mêmes quartiers ou secteurs de résidence que les filles scolarisées. Leur choix fut accidentel.

Nous avons collecté les données par entretien à travers des focus group. Quatre groupes de discussion ont été constitués. Les focus groups étaient animés par deux personnes dont une prenait des notes et l’autre chargée d’animer la discussion. Avant le début de chaque discussion, les participantes étaient informées des buts et objectifs de l’étude. Les enquêteurs les assuraient de la confidentialité des informations puis négociaient leur autorisation pour l’enregistrement de la discussion et la prise de notes.

Au total, 24 adolescentes ont participé aux discussions, 11 non scolarisées et 13 scolarisées. Les discussions se sont déroulées en français et en langue nationale dioula et ont duré en moyenne 120 minutes. Toutes les discussions ont été enregistrées sur cassettes, puis retranscrites, préparées, traitées et analysées à l’aide du logiciel QSR Nvivo 2.0.

## Résultats

### Les confidents et les raisons de leurs choix par les adolescentes

Selon les adolescentes, scolarisées ou non, leurs confidents sont la mère, les aînés (grands frères ou grandes sœurs), la tante et les amies. Aucune adolescente n’a cité le père comme confident. Les raisons du choix des personnes citées sont essentiellement la confiance portée à cette personne *« si je veux parler de quelque chose de sérieux, je vais vers mon amie en qui j’ai confiance. »* (FG_AS), les conseils que peut donner cette personne *« c’est aussi à ma mère que je le dis; oui je le dis, elle peut bien me conseiller »* (FG_AS), et la compréhension surtout auprès des ainées et des amies *« je discute le plus souvent avec mon amie parce qu’on se comprend et on a beaucoup de choses à se dire »* (FG_AS.). La peur aussi de voir son secret divulgué amène certaines adolescentes à se confier à leur proche notamment la mère *« parce que si tu le dis au dehors, ça peut se propager, c’est pourquoi je préfère ma mère. Là s’il y a des conseils à prodiguer, elle me les dit »* (FG_ANS)

### Thèmes de discussion avec les parents/tuteurs

Les adolescentes discutent des grossesses non désirées et des IST/VIH/Sida avec leurs parents. Il s’agit de parents qui savent que les adolescentes sont à risque et cherchent à leur éviter les situations de grossesses ou de maladies. Seulement il ne leur est pas fait une éducation sexuelle conséquente. La plupart du temps il s’est agi de conseils ou des mises en garde sur la fréquentation des garçons.

*« Ils me disent de faire attention et de ne pas céder au vagabondage. Il y a une maladie grave (banadjugu). C’est à cause de tout ça que je ne fais plus comme avant »* (FG_ANS)

*« Nous discutons le plus souvent des règles et des questions de grossesse. Elle me dit souvent d’être prudente envers les hommes, de me méfier d’eux en ne cédant pas aux avances qu’ils me font » (FG_ANS). Les questions de menstrues sont souvent évoquées « on parle des règles elle me demande si j’ai vu…Et elle dit si j’ai vu un jour de ne pas avoir peur »* (FG_AS).

*« Elle dit de ne (…) de ne pas aimer, écouter les gens, et puis de ne pas aimer se rapprocher trop des garçons; parce que, euh, personne ne connaît ce qu’ils veulent réellement de toi » FG-AS, « Elle a dit que, euh, euh… il y’a une qu’elle connaît, qui vient des sous vêtements, elle dit qu’il y’a une maladie qui vient des dessous comme le trichomonas.»* (FG_AS).

*« Oui. Moi, je lui demande comment les gens tombent enceinte? Les filles? Et puis, elle me dit il y’a des filles qui aiment s’approcher des garçons; et pendant les rapports sexuels elles ne se protègent pas »* (FG_AS).

Les sujets de discussion montrent le besoin d’information des adolescentes sur des questions aussi importantes que celles de leur santé sexuelle et reproductive. Les pères ne participent pas beaucoup à ces échanges. Les adolescentes non scolarisées semblent discutés le plus avec leurs parents que celles scolarisées. Ceci pourrait se comprendre par le fait qu’elles passent beaucoup plus de temps ensemble surtout celles qui aident pour le ménage et la cuisine ou qui vendent au marché auprès de leur mère.

Les moments de discussion avec les parents ne varient pas selon les adolescentes. Les moments privilégiés pour les échanges sont la nuit, au cours du repas ou après. Ce sont ces moments que les adolescentes choisissent pour poser leurs questions ou leurs inquiétudes.

Dans l’ensemble, les adolescentes apprécient positivement les occasions d’échanges qu’elles ont avec leurs parents ou tuteurs « la discussion que nous avons faite sur les règles m’a beaucoup plu» (FG_AS). « C’était intéressant parce qu’il y’a d’autres mêmes qui veulent que leurs parents, causent avec eux. Et moi, j’ai eu la chance que mes parents causent avec moi; donc, je trouve intéressant cette façon de faire ». (FG_AS).

*Certaines adolescentes souhaitent même plus d’opportunités d’échanges avec leurs parents « Ça m’a plus parce que, euh, euh, c’était ma première fois, de parler des IST avec ma maman. Je peux dire que c’est très bien. Puis, j’aimerais aussi que ça se reproduise encore, parce que j’apprends à connaître bien mes parents » (FG_AS). Des expériences d’échange que les adolescentes ont avec leurs parents, il ressort que les échanges ne sont pas courants et sont de réels moments de bonheur et de joie pour les adolescentes surtout lorsqu’il s’agit du père « ça m’a plus parce que c’était la première fois que… c’était la première fois que je me suis entretenu avec mon père, et puis il n’y’a pas eu de problème. On a bien causé ensemble, et puis c’est le jour aussi qu’il m’a offert un cadeau.»* FG_AS. Les occasions d’échanges sont le plus souvent créées par les parents lorsque ceux-ci sont dans certaines dispositions d’esprit *« En fait c’était un jour, elle était contente, elle m’a fait venir auprès d’elle, et nous avions bien échangé. C’était les conseils qu’elle me donnait »* FG_AS.

Les suggestions des adolescentes pour améliorer la communication avec leurs parents portaient sur l’entente, la cordialité, la paix dans la famille. Ceci semble vraiment important pour les adolescentes et elles l’expriment de la sorte *« On ne veut plus de bagarre dans la famille, parce que dans toute famille il ne faut pas se sous-estimer; parce que c’est toujours des bagarres, des disputes, c’est Dieu qui donne longue vie à l’homme sinon…Même si c’est, quelques fois; deux fois par jour; qu’on se retrouve dans la maison. Se retrouver pour causer, parler de son enfance, de comment on a vécu. Il faut que tout le monde s’entende dans la famille. Ne pas se disputer dans la famille»* FG_AS.

Des valeurs comme la confiance, la franchise, la vérité sont citées par les adolescentes comme pouvant contribuer à améliorer la communication avec les parents. *« Je propose la paix entre nous; de nous comprendre, et puis de dire toujours la vérité »; « il faut que les uns et les autres soient franc »; « on doit se donner des conseils, taire les querelles intestines, être l’un à l’autre des confidents»* (FG_ANS).

### Sujets de discussion avec les amies

Les adolescentes se confient à leurs amies et discutent beaucoup avec elles. Les questions relatives à la physiologie de la femme, les menstrues, la grossesse, les rapports sexuels sont les sujets de prédilection. *« Nous parlons de la physiologie de la femme. »* (FG_AS); *« On parle des règles, de la grossesse »* (FG_AS), *« Quand tu es en règles, qu’est ce que tu dois faire»* (FG_AS). Les sujets de discussion avec les amies sont plus diversifiés qu’avec les parents. Elles discutent plus aisément de grossesse, de menstrues, de rapports sexuels, etc…

*« Nous parlons le plus souvent des cas de grossesses. Elle dit que la plupart du temps de nombreuses adolescentes tombent enceinte. Elle me dit aussi que le plus souvent le début de la grossesse se manifeste par des vomissements, les nausées et l’arrêt des règles. Donc une fois que vous commencez à observer une telle situation, alors dites-vous que c’est une grossesse »* (FG_ANS).

*« Pendant nos causeries, nous nous disons que si quelqu’un d’entre nous doit avoir des rapports sexuels, que la personne se protège. Il faut qu’on fasse attention à notre santé »*. (FG_AS).

En termes de satisfaction des échanges avec les amies, les avis des adolescentes sont partagés. Certaines estiment qu’elles sont satisfaites des échanges, d’autres non. Les amies n’ont pas toujours des réponses aux questions et elles doivent se référer à d’autres personnes *« Je ne suis pas totalement satisfaite parce qu’elle ne peut pas répondre à toutes mes questions. Je pars demander à quelqu’un d’autre, à ma mère ou à ma sœur»* FG_AS.

### Communication et facteurs de risques des IST/VIH/SIDA et grossesses indésirées Perceptions et expériences de la sexualité des adolescentes

Toutes les adolescentes interrogées disent n’avoir pas encore eu de rapports sexuels. Mais la question de la sexualité n’est pas véritablement abordée par les adolescentes avec leurs parents. Le sujet semble difficile à discuter avec ces derniers. Aucune adolescente n’a avoué son expérience en matière de sexualité. Le sujet est très sensible et la gêne entre elles ne favorisait pas vraiment le partage de ce type d’expérience. Néanmoins certaines ont dit avoir déjà vu des films pornographiques et le sentiment qu’elles ont eu est l’étonnement *« j’étais très étonné et je me posais beaucoup de questions »* (FG_ANS).

Les questions concernent aussi bien l’acte sexuel « je demande souvent à mon amie, comment se font les rapports sexuels? Mais elle me répond qu’elle ne sait pas donc je lui propose de poser la question à quelqu’un d’autre » FG_ANS), la fécondation et l’enfantement *« moi j’aimerai savoir comment on gagne un enfant? »* (FG_ANS), l’avortement *« Je voudrais savoir davantage sur les conséquences de l’avortement? »* (FG_ANS), *« Qu’est ce qui amène les filles à avorter? »* (FG_ANS) le viol *« Je voulais savoir pourquoi les hommes forcent les filles à coucher avec eux?* (FG_AS), l’adultère *« Pourquoi les femmes bien qu’elles soient mariées, elles commettent l’adultère? »* (FG_AS), l’excision « Pourquoi les parents contraignent leurs enfants à l’excision? » (FG_ANS). Toutes ces questions posées sont discutées dans la majorité des cas avec les amies ou les aînées *« (Parce que) avec ma mère, je me gêne beaucoup mais avec ma sœur je ne suis pas aussi gênée que ça »* FG_AS.

Les questions sur les menstrues sont celles qui sont discutées avec les mères. Les adolescentes qui voient déjà leurs règles ont dit l’avoir annoncé à leur mère *« Moi je l’ai dit à ma mère et elle m’a assuré que ce sont les règles »* (FG_ANS). Leurs principales perceptions des règles sont: *« Lorsque tu commences à voir tes règles, c’est ce qui prouves que tu es une femme »* (FG_ANS), *« si tu as par exemple douze (12) ou treize (13) ans comme ça; les règles commencent. Et si ça commence, il faut te laver au moins trois (03) fois par jour, il faut être propre tous les jours »* (FG_AS).

### Connaissances et perceptions des adolescentes des IST-VIH/SIDA

De façon générale, les adolescentes ont des connaissances sur le VIH/Sida. Elles décrivent les modes de transmission et les moyens de prévention. Elles disent discuter du sujet avec leurs parents. Les adolescentes sont très prolixes des modes de transmission *« c’est avec les hommes qu’on peut avoir le sida », « Si quelqu’un a le sida et se coupe avec une lame, si toi à ton tour tu te coupes avec la même lame, il parait que tu peux avoir le sida », « Si une femme tombe enceinte et qu’elle est infectée du sida, si elle ne suit pas un traitement à l’hôpital, à l’accouchement, l’enfant peut être infecté », « Le fer peut amener le sida, l’aiguille peut être aussi à la base comme lorsqu’on se tresse. C’est pourquoi en se tressant, il faut apporter ton aiguille, ta lame sinon si tu utilises la même lame avec quelqu’un qui a le sida, tu peux avoir le sida »*.

Quelles perceptions les adolescentes se font-elles du VIH/Sida? Le Sida reste une maladie redoutable qu’il faut éviter à tout prix *«On dit que c’est une maladie qui ne peut pas se guérir. … mais pour éviter cette maladie, il faut se protéger avec des préservatifs. », « Pour moi, le SIDA, c’est une maladie qu’on ne peut pas soigner alors il faut que tout un chacun prenne ses précautions pour l’éviter »*.

Les adolescentes se disent être à risque car n’ayant pas encore eu de rapports sexuels; elles disent que la voie sexuelle est la voie la plus courante pour l’infection à VIH et de ce fait il est nécessaire de se protéger. Le moyen de prévention cité par les adolescentes est le préservatif. Même celles qui disent avoir discuté de la question avec leurs parents ne citent que le préservatif comme moyen de prévention. *« On dit que c’est une maladie qui ne peut pas se guérir. … mais pour éviter cette maladie, il faut se protéger avec des préservatifs », « elle (mère) dit que pour éviter ces maladies, il faut se protéger avec des préservatifs »*. Le seul moyen de protection cité par les adolescentes qu’elles soient scolarisées ou non est le préservatif. Il n’est fait mention nulle part de l’abstinence (même circonstancielle) ou de la fidélité. Il est important de noter qu’au cours des discussions, des adolescentes ont avoué ne pas savoir ce que c’est que le VIH/sida. Elles n’en ont pas entendu parler ou en discuter avec quelqu’un *« je n’ai jamais entendu parler du sida »*. Le test de dépistage est évoqué comme moyen de savoir si l’on est infecté ou pas mais aucune des adolescentes n’a rapporté avoir déjà fait le test.

### Connaissances et perceptions des méthodes contraceptives par les adolescentes

Les adolescentes ont quelques connaissances des contraceptifs. Elles citent entre autre la pilule *« médicament qu’on avale », le collier, le stérilet, le préservatif, les injections « piqûres »*, la méthode des calendriers. Les adolescentes s’expriment en ces termes *« On dit qu’il y a à l’hôpital son médicament et son plastique (bracelet), sa piqûre et le bracelet qui se porte à la main. »; « il y a certaines femmes qui vont faire des injections, on peut prendre aussi des comprimés, j’ai oublié le nom (…) la pilule »*. Les adolescentes scolarisées disent discuter de ces méthodes contraceptives à l’école avec certains de leurs professeurs. Quant aux non-scolarisées, c’est à travers les causeries avec les autres femmes ou avec leurs amies. Cependant certaines adolescentes non scolarisées disent ne pas connaitre les méthodes contraceptives. Elles n’en ont jamais entendu parler *« non, je n’ai jamais rien entendu de tel »*.

De façon générale, les adolescentes font une bonne appréciation des méthodes contraceptives en ce sens qu’elles permettent de limiter le nombre d’enfants et d’éviter les grossesses non désirées: *« accoucher chaque année, chaque année, chaque année, chaque année, à force d’accoucher, tu auras beaucoup d’enfants, et puis il faut les éduquer et les nourrir, et puis chaque fois, il faut parler, parler, parler, parler, toi-même, tu va devenir, vieille, pendant que tu es encore jeune »*. C’est dire que de ce point de vue, les méthodes contraceptives présentent de nombreux avantages. Néanmoins certaines adolescentes se posent des questions du genre *« pourquoi ce sont les femmes qui utilisent les contraceptifs, et non pas les hommes, à part les préservatifs? »*.

Bien que certaines adolescentes pensent que les méthodes contraceptives sont bien pour limiter les naissances et les souffrances des femmes, d’autres pensent que ces méthodes ont des conséquences sur la santé *« Moi, je pense que, la méthode des médicaments, ce n’est pas bien, parce que si tu te trompes avec les médicaments, …Tu peux faire beaucoup d’enfants », « ça peut rendre malade »; « ça peut rendre stérile », « ça peut te faire grossir »*. Bien qu’elles n’aient pas d’expériences dans l’utilisation de ces méthodes, elles font mention de l’expérience vécue par d’autres personnes dans leur entourage.

## Discussion

La participation des adolescentes aux discussions, n’a pas vraiment permis d’explorer en profondeur certains thèmes. Néanmoins l’information disponible donne déjà des indicateurs sur l’ampleur des problèmes de communication avec les adolescentes qu’elles soient scolarisées ou non. Les résultats montrent que la qualité de la communication parents-adolescentes n’est pas des meilleures pour favoriser une réelle discussion des questions de santé reproductive des adolescentes.

Les adolescentes ne sont pas très informées et n’ont pas aussi l’occasion de discuter de ces questions avec leurs parents. Les questions « sensibles » sont discutées avec les amies ou les aînées qui sont aussi des adolescentes ou à peine sorties de l’adolescence avec les mêmes besoins et les mêmes questions. Ce déficit de communication ne concerne pas seulement les filles; des études faites sur les garçons montrent qu’il y a aussi des problèmes de communication avec les parents surtout sur les questions de la sexualité. Au Sénégal, Nafissatou Diop et collègues [10] ont montré que les parents n’avaient pas une certaine ouverture d’esprit pour entamer une discussion avec leurs enfants sur le sexe. De même, les adolescents se trouvaient très embarrassés de discuter de ces questions avec leurs parents. C’est le cas aussi dans notre étude où les parents n’ont pas le reflexe de discuter ces questions avec les adolescentes. Crosby [11] a montré que les enfants qui avaient une bonne relation avec leurs parents discutaient plus aisément les questions sur la sexualité et entraient plus tardivement dans une activité sexuelle et étaient plus enclins à utiliser le préservatif que ceux qui n’avaient pas de bonnes relations avec leurs parents ou famille. Ne faut-il pas comprendre par ici la nécessité tant évoquée par les adolescentes d’une bonne entente, la cohésion et la paix dans les familles pour l’amélioration de la communication avec les parents?

Dans les familles, les pères ne discutent pas beaucoup avec leurs filles sur leur santé sexuelle et reproductive; ce sont les mères qui s’en chargent avec tout ce que cela peut comporter comme limites et informations erronées vu que les parents de façon générale ne sont pas aussi bien imprégnées de ces questions surtout pour ce qui concernent les besoins des adolescentes. Il est d’autant plus difficile pour notre population d’étude (adolescentes), de commencer du jour au lendemain des discussions avec les parents sur le sexe; cela laisserait croire qu’elles sont déjà actives sexuellement; la peur des parents ne facilite pas ce genre d’initiatives et les adolescentes doivent passer par l’école de la rue pour s’instruire sur la santé sexuelle et reproductive. Les connaissances des adolescentes des infections sexuellement transmissibles et le VIH, ne sont pas des meilleures. Elles reprennent souvent sans trop de conviction les messages reçus sur les modes de transmission. Il en est de même des moyens de prévention. Le préservatif est systématiquement cité, mais pour des adolescentes qui n’ont pas de notions assez claire en matière de sexualité, ne peuvent pas vraiment comprendre toute l’importance de ce moyen de prévention. Aussi, n’ayant pas fait allusion à l’infection à VIH et la possibilité de porteurs asymptomatiques, il reste évident que facilement, elles peuvent tomber dans les travers et ne pas avoir un réel pouvoir de négociation pour l’utilisation du préservatif. Alors, l’on est tenté de se demander si l’abstinence, pour les jeunes filles qui disent n’avoir pas encore eu de rapports sexuels, ne compte pas ou est seulement le fait de l’ignorance. Il est vrai que le préservatif est le moyen de prévention le plus couramment accepté mais nous pensons que pour les adolescentes, l’abstinence reste le moyen le plus efficace et plus sûr au regard des craintes qu’elles ont exprimé pour se protéger contre ces maladies. Il en est de même des grossesses non désirées et précoces; les connaissances des adolescentes des méthodes contraceptives ne sont pas du tout bonnes. Il est important que les adolescentes soient encouragées à entrer le plus tardivement possible dans une activité sexuelle qui peut être à risque au regard de leur jeune âge, leur manque d’expériences et la faible capacité de négociation pour une santé sexuelle et reproductive responsable.

## Conclusion

A la lumière de ce travail, il apparaît nécessaire de doter les parents des compétences nécessaires pour qu’ils puissent aborder les questions de santé reproductive et sexuelle des adolescentes. Ceci est d’autant plus important que le comportement des parents est à l’image de ce qu’ils ont aussi reçu de leurs parents. Les moyens de communications (radio, télévision, internet) véhiculent de nombreuses informations et peuvent être des terreaux d’apprentissage pour les adolescentes, avec touts les risques de mauvaises compréhensions, de mauvaises interprétations. Bien que les médias jouent leur rôle, il est à noter que rien ne pourrait aussi bien faciliter l’adoption de comportements responsables qu’une bonne communication entre parents et adolescentes. Pour ce faire, ne serait-il pas possible de mettre en place une intervention qui travaillerait avec les parents dans le but de leur donner toutes les informations nécessaire sur la santé sexuelle et reproductive, les techniques pour mieux communiquer avec les adolescentes.

## References

[1] Stanley K Henshaw US Teenage Pregnancy Statistics with Comparative Statistics for Women Aged 20–24.

[2] Eaton DK, Kann L, Kinchen S, Ross J (2006). Youth risk behavior surveillance--United States, 2005. MMWR Surveill Summ.

[3] Whitaker DJ, Miller KS, May DC, Levin ML (1999). Teenage partners’ communication about sexual risk and condom use: the importance of parent-teenager discussions. Fam Plann Perspect..

[4] DiIorio C, Kelley M, Hockenberry-Eaton M (1999). Communication about sexual issues: mothers, fathers, and friends. J Adolesc Health..

[5] Biglan A (1990). Social and behavioral factors associated with high-risk sexual behaviour among adolescents. Journal of behavioral medicine..

[6] Small SA, Luster T (1994). Adolescent sexual activity: an ecological, risk-factor approach. Journal of marriage and the family..

[7] DiClemente RJ (2001). Parental monitoring and its association with a spectrum of adolescent health risk behaviours. Pediatrics..

[8] MASSN-MS-UNFPA-Population Council (2002). Etude diagnostic au niveau communautaire sur le vécu des adolescents(es) et de leurs besoins en santé de la reproduction au Bazéga et au Gourma.

[9] MASSN-MS-UNFPA- Population Council-DFID (2002). Revue des politiques et des programmes sur la santé de la reproduction des adolescents au Burkina Faso.

[10] Nafissatou J, Diop Ph D Alioune Diagne: Improving communication between parents and adolescents on reproductive health and HIV/AIDS Frontiers in Reproductive Health.

[11] Crosby RA, Miller KS, Wingood GM, DiClemente RJ Family influences on adolescent females’sexual health.

